# Midline involvement and perineural invasion predict contralateral neck metastasis that affects overall and disease-free survival in locally advanced oral tongue squamous cell carcinoma

**DOI:** 10.3389/fonc.2022.1010252

**Published:** 2022-10-26

**Authors:** Maki Akamatsu, Takuma Makino, Shinya Morita, Yohei Noda, Shin Kariya, Tomoo Onoda, Mizuo Ando, Yoshihiro Kimata, Kazunori Nishizaki, Mitsuhiro Okano, Aiko Oka, Kengo Kanai, Yoshihiro Watanabe, Yorihisa Imanishi

**Affiliations:** ^1^ Department of Otorhinolaryngology–Head and Neck Surgery, Okayama University, Graduate School of Medicine, Okayama, Japan; ^2^ Department of Otorhinolaryngology–Head and Neck Surgery, International University of Health and Welfare, School of Medicine, Narita Hospital, Chiba, Japan; ^3^ Department of Plastic and Reconstructive Surgery, Okayama University, Graduate School of Medicine, Okayama, Japan

**Keywords:** contralateral neck metastasis, contralateral cN0 neck, diagnostic performance, disease-free survival, locally advanced tongue squamous cell carcinoma, midline involvement, overall survival, perineural invasion

## Abstract

**Introduction:**

Although patients with oral squamous cell carcinoma who develop contralateral neck metastasis (CLNM) have worse survival outcomes than those without CLNM, accurate prediction of occult CLNM in clinically negative contralateral neck (contralateral cN0) remains difficult. This study aimed to identify clinicopathological factors that could reliably predict CLNM in patients with locally advanced (clinical T3 and T4a) tongue squamous cell carcinoma (TSCC).

**Patients and methods:**

The medical data of 32 patients with cT3–4a TSCC who underwent curative surgery between 2010 and 2017 were retrospectively analyzed. The correlation of clinicopathological variables with CLNM was examined using logistic regression analysis. The diagnostic performance of significant variables was evaluated using the area under the receiver operating characteristic curves (AUC). Overall survival (OS) and disease-free survival (DFS) were assessed using a Cox proportional hazards model.

**Results:**

CLNM was eventually confirmed in 11 patients (34.4%). Multivariate logistic regression showed that midline involvement [odds ratio (OR) = 23.10, P = 0.017] and perineural invasion (PNI, OR = 14.96, P = 0.014) were independent predictors of CLNM. Notably, the prediction model comprising a combination of midline involvement and PNI exhibited superior diagnostic performance with an even higher OR of 80.00 (P < 0.001), accuracy of 90.3%, and AUC of 0.876. The multivariate Cox hazards model revealed independent significance of CLNM as an unfavorable prognostic factor for both OS [hazard ratio (HR) = 5.154, P = 0.031] and DFS (HR = 3.359, P = 0.038), as well as that of PNI for OS (HR = 5.623, P = 0.033).

**Conclusion:**

Our findings suggest that coexisting midline involvement and PNI of the primary tumor is highly predictive of CLNM development, which independently affects both OS and DFS in patients with locally advanced TSCC. Such reliable prediction enables efficient control of CLNM by optimizing management of the contralateral cN0 neck, which will likely contribute to improved prognosis of those patients without unnecessarily compromising their quality of life.

## Introduction

Neck lymph node involvement is one of the most unfavorable prognostic factors in patients with head and neck squamous cell carcinoma (HNSCC), including oral squamous cell carcinoma (OSCC) (1–4). Because the oral cavity has an extensive submucosal lymphatic plexus, OSCC, even in small tumors such as T1–2 tongue squamous cell carcinoma (TSCC), is prone to micrometastases in the neck lymph nodes (5, 6). In addition, since this rich lymphatic network freely communicates across a fibrous septum that constitutes the midline of the tongue, TSCC that has reached the tongue midline has an increased chance of metastasis to the lymph node in the contralateral side of the neck (5, 6). Although contralateral neck metastasis (CLNM) develops much less frequently than ipsilateral metastasis, patients with OSCC, including TSCC, who have developed CLNM have even worse survival rates (7–10).

In patients with clinical stage I/II (cT1–2N0M0) TSCC who underwent partial glossectomy alone followed by strict observation, 14–48% developed delayed neck metastasis (DNM), mostly in the ipsilateral side, during the follow-up period (11–18). DNM tends to be associated with adverse characteristics, such as extranodal extension and multiple node involvement, thereby resulting in worse survival rates (11–15). Hence, a number of studies have explored histopathological features and molecular biomarkers that can predict DNM in stage I/II TSCC. Reliable results confirmed by multivariate analysis have shown several factors independently correlated with DNM, such as tumor thickness or depth, differentiation, vascular invasion, and the expression of particular proteins in the primary tumor (12, 13, 16–20). Some of these findings are indicators for prophylactic treatment for the ipsilateral neck in patients with stage I/II TSCC, with relatively mature consensus statements and guidelines for elective neck dissection or irradiation for the cN0 neck (21).

In contrast, indications for prophylactic treatment for clinically negative contralateral neck (contralateral cN0) in patients with clinical stage III/IV (≥ T3 and/or ≥ N1, but not cN2c) remain an unresolved issue. Indeed, it is often difficult to judge whether to perform elective neck dissection for the contralateral cN0 neck (i.e., bilateral neck dissection for <cN2c patient), because it also involves a dilemma between improving tumor control and decreasing postoperative function, even in advanced stage patients. In addition, few reports of determinants of CLNM have been corroborated by multivariate analysis (10, 22–24), as compared with those regarding the predictors of ipsilateral nodal metastasis, such as DNM.

Herein, we conducted a retrospective analysis to identify clinicopathological factors that could reliably predict CLNM, including the occult form in contralateral cN0 neck, in patients with locally advanced (clinical T3 and T4a) TSCC. We also evaluated the impact of those variables and CLNM on survival in the same cohort.

## Patients and methods

### Study cohort

Patients who underwent curative surgical treatment for locally advanced (cT3-4a) TSCC, primarily at Okayama University Hospital from August 2010 through August 2017, were considered eligible for inclusion in this study. Those that met at least one of the following criteria were excluded: (1) contra-indication for surgery (unresectable tumor); (2) contra-indication for general anesthesia due to other diseases; (3) presence of distant metastasis or other simultaneous primary cancers at the time of diagnosis; (4) previous history of irradiation to the neck for other diseases; and (5) patients who eventually underwent elective radiotherapy to the untreated contralateral cN0 neck. Detailed clinical, pathological, and therapeutic information of 32 patients, who met the abovementioned criteria and had a minimum follow-up period of 36 months or until the patient’s death, were retrieved from the electronic medical database.

This study was approved by the Institutional Review Board and Research Ethics Committee of our hospital. The requirement for informed consent was waived due to the retrospective nature of the analysis.

### Staging and imaging

The tumor stages were classified according to the American Joint Committee on Cancer TNM staging system (8^th^ edition, 2017). Pretreatment staging was carried out by means of physical examination and imaging diagnosis, including computed topography (CT) and magnetic resonance imaging (MRI), and ^18^F-fluorodeoxyglucose (FDG) positron emission tomography/CT (^18^F-FDG PET/CT) imaging, if necessary. Midline involvement of the primary tumor was defined as positive if the tumor had reached or exceeded the midline of the tongue, which was determined by MRI taken immediately before the definitive surgery.

### Induction chemotherapy

Prior to radical surgery, 27 patients, representing most of the cohort, were given 1–2 cycles of induction chemotherapy (ICT), primarily to prevent further tumor growth and metastasis during the waiting period between diagnosis and surgery that tended to be longer than four weeks. The regimens employed were nedaplatin (CDGP)-fluorouracil (FU) in 14 patients, cisplatin (CDDP)-FU in 11, docetaxel-CDDP-FU (TPF) in one, and weekly docetaxel in one. The other five patients were not considered for ICT because of comorbidities, such as renal or hepatic dysfunction, or a shorter wait for surgery with a less risk of tumor progression.

### Definitive surgery

Excision of the primary lesion was essentially performed *via* the pull-through approach, resulting in two-thirds to subtotal glossectomy in 28 patients, subtotal glossectomy with marginal mandibulectomy in 1; total glossectomy with semi-excision of the tongue base in 1; and total glossectomy with total laryngectomy in 2 patients. To ensure a sufficient surgical margin, additional resection was conducted whenever necessary. Plastic surgeons reconstructed the resulting tissue defects in all patients, using a rectus abdominis free flap in 17 patients, an anterolateral thigh free flap in 14, and a forearm free flap in 1.

The extent of neck dissection was basically planned according to the clinical N stage: unilateral for cN0 and bilateral for cN(+). In some cN(+) patients without contralateral neck involvement, contralateral dissection was withheld in consideration of the restriction of operation time associated with advanced age, worse performance status, and serious comorbidities, which resulted in 17 bilateral dissections and 15 unilateral (ipsilateral) dissections. The types of ipsilateral dissection performed were level I–III (supraomohyoid) in 4 patients; level I–IV in 25; and level I–V in 3, while the types of contralateral dissection conducted were level I–II in 2 patients; I–III in 8; level I–IV in 6; and level I–V in 1.

The histopathological findings, including differentiation, vascular invasion, lymphatic invasion, and perineural invasion (PNI), of the resected primary tumor were assessed. In a few patients, only a small number of parameters had not been examined, which were left as missing data in the analysis.

### Adjuvant radiotherapy

Post-operative radiotherapy (PORT) was delivered, in part at the surgeon’s discretion, to five of the patients who revealed adverse pathological findings, such as a positive margin and extranodal extension. The remaining 27 patients did not receive adjuvant radiotherapy after the initial surgery. The standard dose fractionation schedules were in the range of 50 Gy in 25 fractions to 66 Gy in 33 fractions. None of the patients who underwent unilateral (ipsilateral) neck dissection had received PORT to the untreated contralateral neck.

### Definition of contralateral neck metastasis

In this study, CLNM was defined as contralateral neck lymph node metastasis that was histopathologically proven *via* contralateral neck dissection performed either as initial surgery or as salvage surgery for delayed nodal metastasis after the initial surgery. Of 17 patients who initially underwent bilateral dissection, metastasis in the contralateral neck was found in 6 patients, whereas contralateral nodal relapse developed in 1 patient during the follow-up period. Of 15 patients who initially underwent unilateral (ipsilateral) dissection, contralateral nodal relapse emerged in 4 patients during the follow-up. All patients with contralateral nodal relapse were able to undergo salvage dissection to confirm CLNM histopathologically. Overall, 11 (34.4%) patients in this cohort had CLNM.

### Outcome measures and statistical analysis

Multiple clinicopathological variables [age, sex, clinical T (cT) stage, midline involvement, pathological T (pT) stage, histopathologic differentiation, vascular invasion, lymphatic invasion, and PNI] were examined for correlation with CLNM using univariate logistic regression analysis. Odds ratio (OR) and 95% confidence interval (CI) were calculated. The independent significance of the variables found to be significant in the univariate analysis was further assessed *via* multivariate logistic regression. The diagnostic performance of the variables significantly predicting CLNM was evaluated using a two-by-two contingency table analysis combined with the χ^2^ test and Fisher’s exact test. The area under the curve (AUC) of each variable was determined by receiver operating characteristic (ROC) curve analysis.

Overall survival (OS, events: all deaths) and disease-free survival (DFS, events: any of local, regional, or distant relapse; second primary tongue cancer; or any-cause death) were estimated as oncological endpoints using the Kaplan–Meier method. The time to the event was measured from the date of the initial surgery. The significance of differences in survival probabilities associated with the aforementioned variables, as well as CLNM, was examined by univariate analysis using the log-rank test and the Cox proportional hazards model, with hazard ratio (HR) and 95% CI. The multivariate Cox hazards model further evaluated the independent significance of the variables. P values < 0.05 were considered statistically significant. All statistical analyses were carried out using SPSS Statistics Ver. 28.0.1.0 (IBM) and EXCEL Multivariate Analyses for MAC Ver. 3.0 (Esumi Co., Ltd., Tokyo).

## Results

### Patient characteristics

The demographic and clinicopathological characteristics of the 32 patients in the study cohort are summarized in **Table 1**. The median age was 62 (range, 23–90) years, and the male-to-female ratio was approximately 2:1. More than two-thirds of the patients (22 patients, 68.8%) presented with a cT4a tumor, while nearly two-thirds of those (20 patients, 62.5%) showed midline involvement of the primary tumor. Regarding the pT stage, partially owing to ICT, approximately 40% (13 patients) were down-staged to less than pT3. Nodal metastasis was pathologically positive (pN(+)) in approximately two-thirds of the patients (21 patients, 65.6%) after the initial surgery. The pT and pN stage and several other histopathological factors, as well as midline involvement, corresponded to the post-ICT findings if ICT was administered, implying that ICT was regarded as part of the initial treatment, in combination with definitive surgery.

**Table 1:** Patient characteristics (N = 32).

Variables	N	(%)
Age, y		
≦ 60	17	53.1
> 60	15	46.9
Median (range)	62 (23 - 90)	
Mean ± SD	59 ± 17	
Sex		
Female	11	34.4
Male	21	65.6
cT stage*		
cT3	10	31.3
cT4a	22	68.8
Midline involvement**		
No	12	37.5
Yes	20	62.5
pT stage***		
pT0	1	3.1
pT1	2	6.3
pT2	10	31.3
pT3	4	12.5
pT4a	15	46.9
pN stage***		
pN0	11	34.4
pN1	7	21.9
pN2b	7	21.9
pN2c	3	9.4
pN3b	4	12.5
Differentiation		
Well	14	43.8
Moderate	16	50.0
Unknown	2	6.3
Vascular invasion		
No	22	68.8
Yes	10	31.3
Lymphatic invasion		
No	13	40.6
Yes	18	56.3
Unknown	1	3.1
Perineural invasion		
No	15	46.9
Yes	16	50.0
Unknown	1	3.1
CLNM		
No	21	65.6
Yes****	11	34.4
(at initial surgery	6	18.8)
(as nodal relapse	5	15.6)
Induction chemotherapy		
No	5	15.6
Yes	27	84.4
Neck dissection		
Unilateral (Ipsilateral)	15	46.9
Bilateral	17	53.1
Reconstruction		
Rectus abdominis flap	17	53.1
Anterolateral thigh flap	14	43.8
Forearm flap	1	3.1
Adjuvant RT		
No	27	84.4
Yes	5	15.6

CLNM, contralateral neck metastasis; RT, radiotherapy; SD, standard deviation.

*classified before the initial diagnosis.

**determined by MRI taken just before the initial surgery.

***classified after the initial surgery.

****confirmed by either initial or salvage surgery.

### Contralateral neck metastases

**Figure 1:**
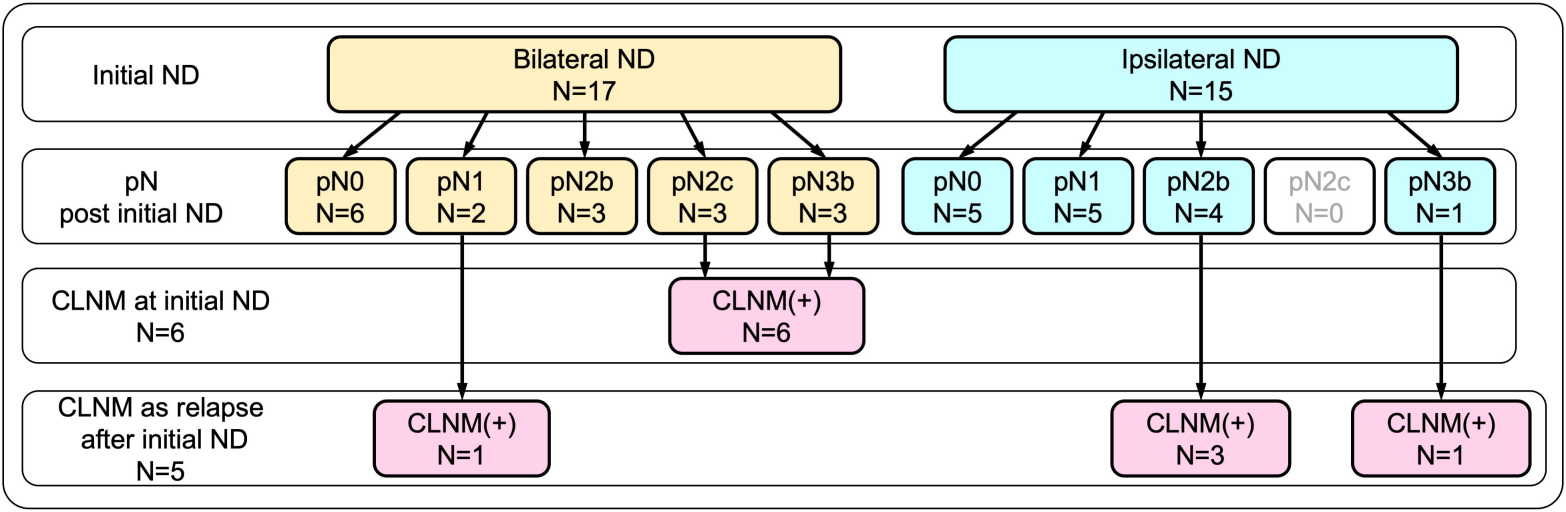
Breakdown of contralateral neck metastasis (CLNM) according to initial neck dissection. CLNM was eventually confirmed in 11 patients (34.4%) by the time of the last follow-up. Of these, six patients were confirmed by bilateral neck dissection performed as part of the initial surgery, while the remaining five patients who developed CLNM as contralateral nodal relapse were confirmed by salvage contralateral neck dissection.

As described above, CLNM was eventually confirmed in 11 patients (34.4%) by the time of the last follow-up. As shown in **Figure 1**, among those, six patients were confirmed to have CLNM by bilateral neck dissection, performed as part of the initial surgery, which included three patients with pN2c and another three patients with pN3b. In the remaining five patients, CLNM developed as contralateral nodal relapse, followed by salvage contralateral neck dissection during the follow-up period. These patients included three patients with pN2b and one patient with pN3b who initially underwent unilateral (ipsilateral) dissection, and one patient with pN1 who initially underwent bilateral dissection.

### Logistic regression analysis of factors affecting CLNM

To determine the factors predictive of CLNM, the association between clinicopathological variables and CLNM was examined using logistic regression analysis. As summarized in **Table 2**, univariate analysis demonstrated that only midline involvement and PNI, and not other variables, were significantly correlated with CLNM. Multivariate analysis confirmed that both midline involvement [OR = 23.100 (95% CI: 1.774–300.938), P = 0.017] and PNI [OR = 14.960 (95% CI: 1.722–129.971), P = 0.014] were independent risk factors predictive of CLNM in this cohort.

**Table 2:** Univariate and multivariate logistic regression analysis for factors affecting CLNM.

Variables		N	CLNM		Univariate analysis					Multivariate analysis				
			Yes (N = 11)	No (N = 21)	OR	95% CI			P value	OR	95% CI			P value
Age, y														
	≦ 60	17	6	11	1.000	reference								
	> 60	15	5	10	1.320	0.305	−	5.704	0.710					
Sex														
	Female	11	5	6	1.000	reference								
	Male	21	6	15	2.083	0.456	−	9.508	0.343					
cT stage														
	cT3	10	2	8	1.000	reference								
	cT4a	22	9	13	2.769	0.473	−	16.213	0.259					
Midline involvement														
	No	12	1	11	1.000	reference				1.000	reference			
	Yes	20	10	10	11.000	1.187	−	101.979	0.035	23.100	1.774	−	300.938	0.017
pT stage														
	pT0−3	17	6	11	1.000	reference								
	pT4a	15	5	10	0.917	0.212	−	3.961	0.907					
Differentiation														
	Well	14	3	11	1.000	reference								
	Moderate	16	8	8	3.667	0.733	−	18.332	0.114					
	Unknown	2	0	2	−				−					
Vascular invasion														
	No	22	6	16	1.000	reference								
	Yes	10	5	5	2.667	0.563	−	12.622	0.216					
Lymphatic invasion														
	No	13	2	11	1.000	reference								
	Yes	18	8	10	4.400	0.749	−	25.842	0.101					
	Unknown	1	1	0	−				−					
Perineural invasion														
	No	15	2	13	1.000	reference				1.000	reference			
	Yes	16	8	8	6.500	1.094	−	38.633	0.040	14.960	1.722	−	129.971	0.014
	Unknown	1	1	0	−				−	−				−

CLNM, contralateral neck metastasis; OR, odds ratio; CI, confidence interval.

### Diagnostic performance of factors for prediction of CLNM

**Figure 2:**
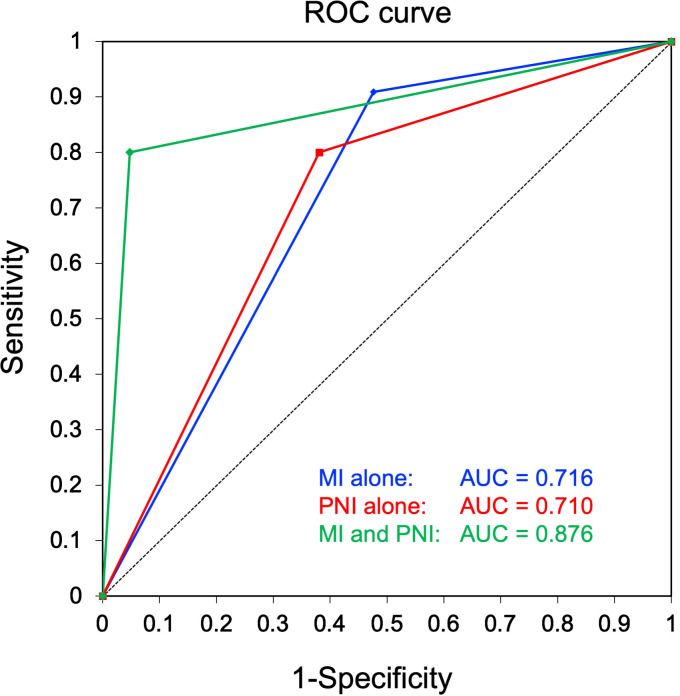
Receiver operating characteristic (ROC) curves for comparison of diagnostic performance in prediction of contralateral neck metastasis. A combination of midline involvement and perineural invasion (PNI), wherein the high-risk group was defined as showing both midline involvement and PNI, while the low-risk group comprised the remaining patients without midline involvement and/or PNI, showed a superior area under the curve (green, 0.876) to that of midline involvement alone (blue, 0.716) and PNI alone (red, 0.710).

To compare the clinical usefulness of each of the identified independent predictive factors as well as their combination in predicting CLNM, the accuracy, sensitivity, specificity, positive-predictive value, negative-predictive value, OR, and AUC were calculated, as summarized in **Table 3**. Notably, in the group of patients positive for both midline involvement and PNI, 8 out of 9 (88.9%) developed CLNM, while only 2 out of 22 (9.1%) patients in the remainder group developed CLNM. This difference was statistically significant (OR = 80.000 [95% CI: 6.331–1010.951], P < 0.001), with excellent accuracy (90.3%) and a superior AUC (0.876; **Figure 2**).

**Table 3:** Diagnostic performance of factors for prediction of CLNM.

Variables	N	CLNM		CLNM incidence	P value		Accuracy	Sensitivity	Specificity	Positive-predictive value	Negative-predictive value	OR	AUC
		Yes (N = 11)	No (N = 21)		χ^2^ test	Fisher’s test							
Midline involvement													
Yes	20	10	10	50.0%	0.016	0.023	65.6%	90.9%	52.4%	50.0%	91.7%	11.00	0.716
No	12	1	11	8.3%									
Perineural invasion													
Yes	16	8	8	50.0%	0.029	0.054	67.7%	80.0%	61.9%	50.0%	86.7%	6.50	0.710
No	15	2	13	13.3%									
Unknown	1	1	0	–									
MI and PNI													
MI(+) and PNI(+)	9	8	1	88.9%	< 0.001	< 0.001	90.3%	80.0%	95.2%	88.9%	90.9%	80.00	0.876
Others	22	2	20	9.1%									
Unknown	1	1	0	–									

CLNM, contralateral neck metastasis; MI, midline involvement; PNI, perineural invasion; OR, odds ratio; AUC, area under the curve.

### Oncological outcomes

The median and mean follow-up of the whole study cohort (n = 32) were 33 and 34 months, respectively, whereas those of the patients alive at the time of the analysis (n = 19) were 46 and 49 months, respectively. During the follow-up period, 12 patients (37.5%) died of the disease, and 1 patient (3.1%) died of another cause (sepsis). At the last follow-up, 17 patients (53.1%) were alive and the disease-free (including 5 patients who underwent secondary salvage treatment for relapse, and remained recurrence-free thereafter), and 2 patients (6.3%) were alive with the disease.

### Survival analysis of factors predictive of OS and DFS

The association of clinicopathological variables with OS and DFS was examined using the Cox proportional hazards model, as summarized in **Table 4**. In univariate analysis, patients with midline involvement showed significantly worse OS (P = 0.040) and DFS (P = 0.033) than those without midline involvement. Similarly, those with CLNM demonstrated significantly poorer OS (P = 0.003) and DFS (P = 0.007) than those without CLNM. Furthermore, PNI was significantly correlated with worse OS (P = 0.028), but not with worse DFS. No other factor was associated with survival.

**Table 4:** Univariate and multivariate Cox regression analysis for factors predictive of overall survival and disease-free survival.

Variables		N	Overall survival						Disease-free survival						
			Univariate analysis			Multivariate analysis*			Univariate analysis			Multivariate analysis*		
			HR	95% CI	P value	HR	95% CI	P value	HR	95% CI	P value	HR	95% CI	P value
Age														
	≦ 60	17	1.000	reference		1.000	reference		1.000	reference		1.000	reference	
	> 60	15	1.390	0.465 − 4.151	0.555	1.814	0.455 − 7.233	0.399	1.148	0.416 − 3.171	0.790	0.773	0.269 − 2.223	0.633
Sex														
	Female	11	1.000	reference		1.000	reference		1.000	reference		1.000	reference	
	Male	21	1.228	0.378 − 3.995	0.733	5.085	0.985 − 26.249	0.052	1.112	0.379 − 3.266	0.846	1.271	0.424 − 3.808	0.669
cT stage														
	cT3	10	1.000	reference					1.000	reference				
	cT4a	22	2.737	0.606 − 12.361	0.191				1.876	0.528 − 6.659	0.331			
Midline involvement														
	No	12	1.000	reference		1.000	reference		1.000	reference		1.000	reference	
	Yes	20	3.897	1.067 − 14.231	0.040	3.004	0.664 − 13.582	0.153	3.496	1.104 − 11.072	0.033	2.476	0.711 − 8.621	0.154
pT stage														
	pT0-3	17	1.000	reference					1.000	reference				
	pT4a	15	2.325	0.699 − 7.736	0.169				2.144	0.718 − 6.407	0.172			
Differentiation														
	Well	14	1.000	reference					1.000	reference				
	Moderate	16	2.932	0.792 − 10.859	0.107				2.281	0.712 − 7.307	0.165			
	Unknown	2	−		−				−		−			
Vascular invasion														
	No	22	1.000	reference					1.000	reference				
	Yes	10	2.419	0.803 − 7.290	0.117				1.325	0.471 − 3.724	0.594			
Lymphatic invasion														
	No	13	1.000	reference					1.000	reference				
	Yes	18	2.197	0.670 − 7.212	0.194				1.282	0.430 − 3.827	0.656			
	Unknown	1	−		−				−		−			
Perineural invasion														
	No	15	1.000	reference		1.000	reference		1.000	reference				
	Yes	16	4.293	1.171 − 15.732	0.028	5.623	1.151 − 27.474	0.033	2.664	0.833 − 8.518	0.099			
	Unknown	1	−		−	−		−	−		−			
CLNM														
	No	21	1.000	reference		1.000	reference		1.000	reference		1.000	reference	
	Yes	11	5.482	1.756 − 17.114	0.003	5.154	1.162 − 22.862	0.031	4.255	1.488 − 12.163	0.007	3.359	1.069 − 10.556	0.038

*Adjusted by age and sex as possible confounders.

CLNM, contralateral neck metastasis; HR, hazard ratio; CI, confidence interval.

**Figure 3:**
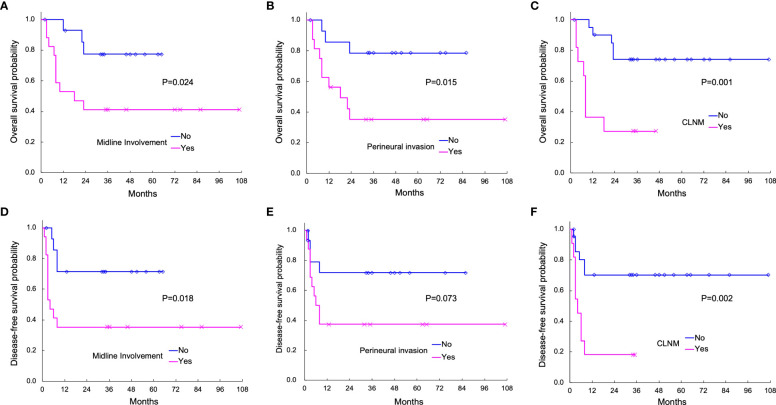
Actuarial Kaplan–Meier survival curves of overall survival (OS; **A–C**) and disease-free survival (DFS; **D–F**) analyzed with the log-rank test. OS according to midline involvement (P = 0.024), perineural invasion (PNI; P = 0.015), and contralateral neck metastasis (CLNM; P = 0.001) is shown in **(A-C)**, respectively. DFS according to midline involvement (P = 0.018), PNI (P = 0.073), and CLNM (P = 0.002) are displayed in **(D-F)**, respectively.

Kaplan–Meier curves of OS according to midline involvement, PNI, and CLNM are displayed in **Figures 3A–C**. The 3-year OS rates with and without midline involvement were 41.2% and 77.4%, respectively (A, P = 0.024), those with and without PNI were 35.2% and 78.6%, respectively (B, P = 0.015), and those with and without CLNM were 27.3% and 74.1%, respectively (C, P = 0.001). Kaplan–Meier curves of DFS according to the same variables are shown in **Figures 3D–F**. The 3-year DFS rates with and without midline involvement were 35.3% and 71.4%, respectively (D, P = 0.018), those with and without PNI were 37.5% and 71.8%, respectively (E, P = 0.073), and those with and without CLNM were 18.2% and 70.2%, respectively (F, P = 0.002).

Multivariate Cox regression analysis adjusted by age and sex (**Table 4**) revealed the independent significance of CLNM as an unfavorable prognostic factor in both OS [HR = 5.154 (95% CI: 1.162–22.862), P = 0.031] and DFS [HR = 3.359 (95% CI: 1.069–10.556), P = 0.038], as well as that of PNI in OS [HR = 5.623 (95% CI: 1.151–27.474), P = 0.033]. Notably, midline involvement was not independently significant in either OS or DFS in multivariate analysis, which remained the same with and without adjustment by age and sex.

## Discussion

Since the oral tongue is involved in several vital functions, including mastication, swallowing, articulation, and tasting, the loss of tongue morphology due to radical resection inevitably causes postoperative dysfunction, thereby leading to impaired quality of life (QOL). In general, while partial glossectomy, which is usually applied in T1–2 TSCC patients, results in relatively minor dysfunction, resection of two-thirds or more of the tongue, as performed in the patients in this study, unavoidably imposes considerable disability on the patients. Hence, in the clinical practice related to locally advanced TSCC, more extended resection for better tumor control always tends to conflict with better functional preservation, even if combined with adequate reconstruction.

Although the procedure of neck dissection has evolved to be more selective and less harmful over a period of decades, long-term morbidities, e.g., persistent lymph edema and lateral head inclination, still develop in a substantial percentage of patients (25). In addition, neck dissection also causes a negative impact on swallowing function, such as forward and downward displacement of the hyoid bone and a decrease in the distance traversed by the hyoid bone (26), although its extent is usually subclinical and less morbid than that caused by resection of the primary site. Hence, adding neck dissection to extended tongue resection, particularly in a form of bilateral dissection, further enhances the degree of postoperative swallowing dysfunction. This indicates that the balance between risk and benefit needs to be considered when judging whether to perform elective neck dissection on the contralateral cN0 neck. Unfortunately, despite great advances in clinical imaging techniques, including ultrasound, CT, MRI, and PET-CT, to date, no diagnostic modalities have been capable of accurately detecting microscopic nodal metastases. Therefore, for efficient selection of patients at high-risk of CLNM as appropriate candidates for prophylactic contralateral neck dissection, it is of utmost importance to identify the clinicopathological characteristics that can reliably predict the occult existence and/or later development of CLNM.

Previous studies have reported an incidence of CLNM in OSCC ranging from 0.9% to 36% (9, 10, 22–24, 27–31). Such wide variance seems to be attributable primarily to differences in patient eligibility criteria in terms of anatomic subsites, location, and stage of the tumor, as well as disparity in standard treatment strategies and histopathological processing at each institution. In this study of TSCC, the incidence of CLNM was as high as 34.4% (11 of 32 patients), presumably because the subjects were limited to locally advanced stage cT3–4a tumors. In line with this, among previous studies that confined the subjects to laterally arising TSCC, a study that screened all stages using a population-based cancer registry, in which cT1–2 constituted 70% of the cohort, showed a very low CLNM incidence of 2.5% (10). In contrast, a study that exclusively analyzed patients with primary tumors that crossed the midline, in which cT3–4 constituted 82% of the cohort, reported a relatively high CLNM incidence of 29.2% (24).

Previous studies have reported several clinicopathological factors associated with CLNM in TSCC (9, 10, 22–24). However, no consensus has been reached on these factors, likely because the eligibility criteria for inclusion were inconsistent across the studies, complicating comparisons among studies much more than in studies regarding ipsilateral neck metastases in early T stage cases. This study suggested that midline involvement and PNI of the primary tumor were able to predict the presence of CLNM, including in occult form, in locally advanced TSCC. In particular, the prediction model composed of a combination of midline involvement and PNI, in which the high-risk group was defined as showing both midline involvement and PNI, while the low-risk group was negative for midline involvement and/or PNI, exhibited a superior diagnostic performance than a model composed solely of midline involvement or PNI. Our finding concerning midline involvement was consistent with two previous studies supported by multivariate analysis. Kowalski et al. demonstrated that, in OSCC, the risk of CLNM in cases of tumors crossing the midline by > 1.0 cm was significantly higher than that of tumors staying > 1.0 cm distant from the midline (OR = 8.8) (22). Lloyd et al. showed that, in laterally arising TSCC, tumors extending across the midline were significantly more likely to involve the contralateral lymph nodes than those not extending to the midline (OR = 9.6) (10). In addition, although lacking multivariate analysis, Koo et al. reported that the rate of CLNM in OSCC was significantly higher in cases of tumors crossing the midline than in those with unilateral lesions (9).

In contrast, Kurita et al. did not find midline involvement to be an independent predictor of CLNM, but instead identified T stage as one of the independently significant predictors (23). The difference between this and the abovementioned studies may be related to the strong correlation found between T stage and midline involvement, indicating that these factors were confounded with each other in the study of Kurita et al. (23). Although Lloyd et al. also reported T stage as an independent predictor of CLNM, in addition to midline involvement, this may be explained at least in part by their markedly larger cohort size, yielding much greater statistical power, than those of other studies (10). In our study, neither cT nor pT stage was identified as a significant predictor, even in univariate analysis, which may be because our inclusion criteria did not include locally early (cT1–2) disease at the time of diagnosis, as well as our relatively smaller cohort.

Three different routes were previously projected for CLNM in HNSCC. The first route involves dissemination from the primary tumor through preexisting midline-crossing afferent lymphatic vessels. The second one is implicated in actual spread beyond the midline from the ipsilateral involved lymph node *via* efferent collateral lymphatic flow that emerges when the ipsilateral lymph nodes become extensively involved. The third one is provided by a primary tumor arising in or invading a central area where there is no real midline barrier (31). These presumptive mechanisms appear to support the critical role of midline involvement in the prediction of CLNM development. Whereas the histopathological information including PNI becomes available only after the initial surgery, it is possible to determine earlier whether the midline is involved. Therefore, midline involvement can predict CLNM both at the time of the initial surgery and in the form of contralateral nodal relapse during the follow-up period, while the predictive value of PNI is limited to the latter.

PNI is histopathologically defined as “tumor cells within any of the three layers (the epineurium, the perineurium, and the endoneurium) of the nerve sheath, or tumor foci outside the nerve with involvement of > 33% of the nerve’s circumference” (32). Because such findings reflect the ability of cancer cells that leave the primary tumor to migrate toward, along, and into the nerve, PNI is considered as another mechanism of tumor spreading, using nerves as the route (32–34).

Several studies using multivariate analysis have demonstrated independently significant association of PNI with the pathological N status (pN), as well as with regional recurrence, in various stages of OSCC (35–38). Another multivariate analysis revealed PNI as an independently significant predictor of occult lymph node metastasis (pN(+) in cN0) in TSCC at the time of diagnosis (39). Regarding association with CLNM, in agreement with these results, our study revealed that PNI is independently correlated with CLNM in locally advanced TSCC.

Although previous studies have not reported independent association between PNI and CLNM, univariate analysis in a previous study showed that the presence of PNI was correlated with a higher risk of CLNM in OSCC patients (22). In terms of predicting contralateral regional relapse in OSCC, only one multivariate analysis found PNI to be an independent predictor of contralateral lymph node recurrence of stage IV A to IV B, well-lateralized OSCC that excluded TSCC (27). Another univariate analysis reported significant association between PNI and contralateral regional relapse in OSCC primarily arising from the lateral side of the oral cavities (40).

Recent basic studies have revealed that PNI involves reciprocal signaling interactions between tumor cells and nerves, wherein invading tumor cells obtain the ability to respond to proinvasive signals within the peripheral nerve environment, while nerves send neurites toward cancer cell colonies in response to their acquired neurotropism (32, 34, 41, 42). Such crosstalk required for PNI is regulated *via* specific molecular signals that initiate and drive processes including neuritogenesis, which is mediated by various neurotrophic factors, such as nerve growth factor (NGF), brain-derived neurotrophic factor (BDNF), and glial cell line-derived neurotrophic factor (GDNF) (32–34, 41–43). Furthermore, cancer–nerve crosstalk was found to represent a mechanism by which loss of p53 function drives reprogramming of tumor-associated neurons toward an adrenergic phenotype that stimulates tumor progression, and thus can be a potential target for anticancer therapy (44). Accordingly, PNI functions as an additional mechanism through which tumor cells spread well beyond the extent achieved by direct local invasion, which facilitates infiltration into contralateral afferent lymph vessels independently of direct lymphatic invasion and midline involvement, thereby increasing the likelihood of CLNM.

Multivariate survival analysis in the present study showed that CLNM is independently correlated with both OS and DFS, while PNI is independently associated with only OS, in patients with locally advanced TSCC. Intriguingly, although midline involvement was found to be significantly correlated with both OS and DFS in univariate analysis, it was not independently correlated with either OS or DFS in multivariate analysis. Considering the limited sample size of this study, such a result appears reasonable because, as shown in the above results, midline involvement is independently associated with CLNM, i.e., they are mutually confounding in this cohort. Thus, midline involvement appears to worsen OS and DFS by promoting CLNM development.

Our result regarding the impact of CLNM corroborates the findings of the few previous studies that reported its adverse effects on survival outcomes in OSCC. An earlier study reported a lower 5-year cure rate in patients with than in those without bilateral node involvement, although the study lacked statistical confirmation (7). In subsequent studies, OS and DFS in OSCC patients with CLNM were shown to be significantly worse than those with no CLNM (8), while OSCC patients with CLNM showed significantly lower OS and disease-specific survival (DSS) than did those without CLNM (9), although both of these results were limited to univariate analysis. A later multivariate Cox regression analysis of a larger TSCC cohort found that CLNM was one of the independent prognostic factors of OS, as were midline involvement, T2 stage, male sex, older age, and high histological grade (10).

Concerning the impact of PNI on survival in OSCC patients, a number of recent studies using multivariate analysis reported its independently significant association with a worse OS (45–47), DFS (47–49), and DSS (37, 46, 50, 51), as well as with increased recurrence at local (45, 50), regional (36, 37), loco-regional (48), and distant (51) sites. The prognostic value of PNI for OS observed in our study was also in accordance with these results, some of which were further validated by recent meta-analyses (52, 53).

In our cohort of locally advanced TSCC, both CLNM and PNI appear to affect OS independently of each other. In contrast, the results for DFS, for which the only independent prognostic factor was CLNM, seems attributable to the low statistical power, and does not necessarily reflect the true cause–effect relationship in this cohort well. Considering that DFS generally reflects all patterns of relapse in local, regional, and distant sites and that CLNM is basically implicated in a regional relapse, it is likely that not only CLNM, but also midline involvement, PNI, and other possible factors, contribute to worse DFS by cooperating to promote various patterns of recurrence.

Based on the present results, among locally advanced TSCC patients with a contralateral cN0 neck, those who have midline involvement preoperatively and/or show PNI postoperatively should be considered to have an increased risk of CLNM, suggesting that they would likely benefit from elective contralateral neck dissection. In case elective contralateral dissection is withheld, prophylactic irradiation to the contralateral neck can be considered; alternatively, very close follow-up is mandatory for those patients. Such reasonably selective treatment for the patients who are reliably assessed to be at high risk of CLNM may eventually improve their OS and DFS.

The present results should be interpreted in the light of several limitations. First, the study involved an inherent selection bias due to its retrospective nature. For example, because of the exclusion criteria, patients with unresectable tumor and/or distant metastasis at the time of diagnosis, who did not undergo curative treatment, were not included in the study, which may have underestimated the incidence of CLNM development. Second, due to the relatively small sample size, the statistical power may not have been sufficient to reveal the significance of other possible predictive factors. However, since the likelihood of CLNM in early T stage is presumed to be much lower than that in advanced T stage, from a clinical perspective, it is worth elucidating predictors of CLNM exclusively in patients with cT3-4a tumors. Third, because ICT was incorporated into the initial treatment along with the definitive surgery, the incidence of CLNM, as well as clinicopathological findings evaluated immediately before or after the definitive surgery, were possibly underrated as compared to those before or without ICT, depending on the response to ICT. Fourth, since we have not validated our prediction model using an external cohort, a validation study or a prospective trial will be essential to corroborate the reliability of its predictive performance.

## Conclusion

Taken together, the present study showed that coexisting midline involvement and PNI of the primary tumor is highly predictive of CLNM development, which independently affects both OS and DFS in patients with locally advanced TSCC. Thus, efficient control of CLNM by appropriately optimizing management of the contralateral cN0 neck, supported by our reliable prediction model comprising midline involvement and PNI, could contribute to improved prognosis of these patients, without unnecessarily compromising their QOL.

## Data availability statement

The original contributions presented in the study are included in the article/Supplementary Material. Further inquiries can be directed to the corresponding author.

## Ethics statement

The studies involving human participants were reviewed and approved by The Institutional Review Board and Research Ethics Committee of Okayama University Hospital. Written informed consent for participation was not required for this study in accordance with the national legislation and the institutional requirements.

## Author contributions

MAk conceived and designed the study, managed the patients, collected and analyzed the data, and drafted the manuscript. TM and YN supervised the study, managed the patients, and provided essential instruction. SM managed the patients and helped the data collection. SK offered the administrative support and made instructive suggestions. TO manage the patients and gave the administrative support. MAn, YK, KN, and MO provided helpful advice and administrative support. AO, KK, and YW participated in the data interpretation and gave valuable suggestions. YI provided comprehensive support, helped the data analysis and interpretation, and wrote and revised the manuscript. All authors contributed to the article and approved the submitted version.

## Funding

This work was supported in part by the Grants-in-Aid for Scientific Research (C) from The Japan Society for the Promotion of Science (19K09876).

## Acknowledgments

We would like to thank Editage (www.editage.com) for English language editing.

## Conflict of interest

The authors declare that the research was conducted in the absence of any commercial or financial relationships that could be construed as a potential conflict of interest.

## Publisher’s note

All claims expressed in this article are solely those of the authors and do not necessarily represent those of their affiliated organizations, or those of the publisher, the editors and the reviewers. Any product that may be evaluated in this article, or claim that may be made by its manufacturer, is not guaranteed or endorsed by the publisher.
